# Wittig and Wittig–Horner Reactions under Sonication Conditions

**DOI:** 10.3390/molecules28041958

**Published:** 2023-02-18

**Authors:** Gheorghe Ilia, Vasile Simulescu, Nicoleta Plesu, Vlad Chiriac, Petru Merghes

**Affiliations:** 1 Faculty of Chemistry, Biology, Geography, West University of Timisoara, 16 Pestalozzi Street, 300115 Timisoara, Romania; 2 Institute of Chemistry “Coriolan Dragulescu”, 24 Mihai Viteazul Blv., 300223 Timisoara, Romania; 3 Banat’s University of Agricultural Sciences and Veterinary Medicine ‘‘King Michael I of Romania’’ from Timisoara, 119 Calea Aradului, 300645 Timisoara, Romania

**Keywords:** Wittig reaction, Wittig–Horner synthesis, ultrasound, carbonyl olefination

## Abstract

Carbonyl olefinations are among the most important organic syntheses that form C=C bonds, as they usually have high yields and in addition offer excellent stereoselectivity. Due to these advantages, carbonyl olefinations have important pharmaceutical and industrial applications. These reactions contain an additional step of an α-functionalized carbanion to an aldehyde or ketone to produce alkenes, but syntheses performed using metal carbene complexes are also known. The Wittig reaction is an example of carbonyl olefination, one of the best ways to synthesize alkenes. This involves the chemical reaction between an aldehyde or ketone with a so-called Wittig reagent, for instance phosphonium ylide. Triphenylphosphine-derived ylides and trialkylphosphine-derived ylides are the most common phosphorous compounds used as Wittig reagents. The Wittig reaction is commonly involved in the synthesis of novel anti-cancer and anti-viral compounds. In recent decades, the use of ultrasound on the Wittig reaction (and on different modified Wittig syntheses, such as the Wittig–Horner reaction or the aza-Wittig method) has been studied as a green synthesis. In addition to the advantage of green synthesis, the use of ultrasounds in general also improved the yield and reduced the reaction time. All of these chemical syntheses conducted under ultrasound will be described further in the present review.

## 1. Introduction

**Figure 1:**
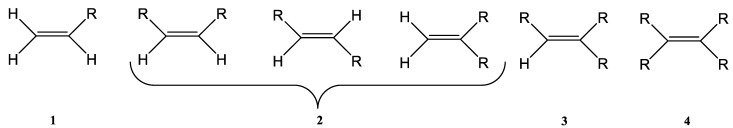
The structure of monosubstituted (**1**), disubstituted (**2**), trisubstituted (**3**), and tetrasubstituted (**4**) alkenes.

The Wittig reaction is a chemical reaction between an aldehyde or ketone and a phosphonium ylide in the presence of a base to provide two compounds: an alkene, which has the position of the double bond well specified, and phosphine oxide. Triphenyl phosphorylide is often referred to as a Wittig reagent. This reaction was discovered in 1954 [1] by Georg Wittig. He received the Nobel Prize in Chemistry in 1979 for its discovery. It is a convenient chemical reaction for the synthesis of alkenes. Usually, disubstituted (cis, trans, or 1,1-disubstituted alkenes, Figure 1) and trisubstituted alkenes can be obtained with good yields, but for tetrasubstituted alkenes the yields are lower. Wittig reactions imply the coupling between aldehydes or ketones with monosubstituted triphenylphosphonium ylides. Interest in obtaining trisubstituted or tetrasubstituted alkenes (Figure 1) has increased in recent decades because of their higher stability in comparison with monosubstituted and disubstituted ones. Furthermore, tri- and tetrasubstituted alkenes are important synthetic targets and occasionally their synthesis can be difficult due to steric issues.

When Wittig synthesis is conducted in classic conditions the yields are rather low, but when using ultrasound, it was observed that the yield increases significantly, especially for obtaining tetrasubstituted alkenes. The Wittig reaction can give as products both E- and Z-alkenes or alkene derivatives. The ratio of E- and Z-isomers could be modified and controlled by changing several parameters (the electronic nature of the ylide carbanion, the presence of lithium salts, the use of phosphonium salt derived either from trialkylphosphine or triarylphosphine) [1,2,3,4,5,6,7,8,9]. The geometry of the double bond is depicted by the nature of the ylide. If the ylides are unstable (they contain an alkyl chain), the reaction product will usually be a Z-alkene. The selectivity of the reaction is moderate. On the other hand, if the ylides are stabilized (for instance by using an ester or a ketone), the product of the reaction will mostly be an E-alkene. The reaction that takes place in this case showed higher selectivity. In the case of semistabilized ylides (they contain an aryl substituent) the selectivity is rarely good and in general a mixture of E-/Z-isomers are obtained with different ratios [1,2,3,4,5,6,7,8,9].

The Wittig mechanism implies hypothetical betaine intermediates and lithium halide adducts. The stereoselectivity of the reaction depends on the formation of the covalent oxaphosphetane and on the result of the combination between steric, rehybridization effects of phosphorous, and on lithium salts [10,11]. In the first step, the alkylation of triphenylphosphine with the halogenated derivative takes place with the formation of a quaternary phosphonium salt. The deprotonation of the phosphonium salt in the presence of the base (as nBuLi, NaNH_2_, NaH, alkoxides, KOH (NaOH), K_2_CO_3_, tertiary amine), produces the phosphorylide (phosphorane). After elimination, if an α-bromoester is used, an enolate ion equivalent is obtained as the phosphorylide is stabilized by conjugation. If the alkylation of triphenylphosphine is undertaken with α-chloroether, a vinyl ether is formed. This undergoes acid hydrolysis. Then the reaction of phosphorylide (the nucleophilic reactant) with the carbonyl compound takes place with the formation of an oxaphosphetane intermediate with a four-atom ring, which, by elimination, leads to the formation of the two stereoisomeric alkenes. In order to obtain the more stable alkene, a phosphorylide stabilized with electron-withdrawing groups, such as vinyl, phenyl, or ester, should be used. For the less-stable alkene, a phosphorylide not stabilized by conjugation is recommended. The obtained Z:E ratios, can be better explained as a function of the level of stabilization (charge delocalization) of the ylide by a mechanism based on a cycloaddition process [12,13].

An important application of the Wittig reaction with industrial importance is the synthesis of juvenile hormones, vitamin A, β-carotene, or other aromas and flavors [14]. In Wittig syntheses, various phosphorus reagents can be used. The ‘‘classic’’ Wittig reaction uses a phosphonium ylide, the Horner–Wadsworth–Emmons reaction uses a phosphonate anion, and the Horner–Wittig uses a phosphine oxide anion [15,16]. For a nitrogen analogue of a Wittig reagent, phosphazenes (λ^5^ -phosphazenes, iminophosphoranes, or phosphine imines) are used in the aza-Wittig reaction [17].

The Wittig and aza-Wittig syntheses become powerful tools in organic synthesis and allow the construction of some acyclic and cyclic compounds. These reactions produce a high yield and require mild conditions (neutral solvents, absence of catalysts, generally at mild temperatures) [18,19].

The Wittig syntheses have been strongly developed and improved during recent decades. Several extensions of the method recently developed are the phospha-Wittig reaction [20], the thia-Wittig reaction [21], and the phospha-bora-Wittig reaction [22]. The phospha-bora-Wittig reaction is used for the direct preparation of phosphaalkenes, starting with aldehydes, ketones, esters, or amides. The intermediate phosphaborene reacts with the carbonyl compounds to form 1,2,3- phosphaboraoxetanes, which then undergoes thermal or Lewis acid-promoted cycloreversion, leading to the formation of phosphaalkenes [22].

A simple search on www.webofscience.com performed on 10 January 2023 for the period 2012–2022 showed that more than 12,000 papers were published in this area. Given that many protocols for the preparation of organic derivatives suffer from limitations such as long reaction times, yields, and selectivity, it is necessary to investigate the power of ultrasound in the promotion of these kinds of reactions.

## 2. Wittig Reactions under Sonication Conditions

The organophosphorus derivatives represent an important class of chemical compounds due to their numerous applications in several fields of great interest. The organophosphorus derivatives are involved in several reactions and syntheses, for example, the Wittig and Horner–Wadsworth–Emmons olefinations, the Arbuzov synthesis, or the Staudinger reaction. The organophosphorus derivatives have applications as active pharmaceutical ingredients and agrochemicals. In addition, the phosphorus-containing compounds are used among others, such as ligands for organometallic complexes, as precursors for products with flame retardant properties and for obtaining organic–inorganic hybrids (metal–organic frameworks) and surface grafted materials (including the use of ultrasound for their synthesis) [1,2,3,4,5,6,7,8,9,23,24,25,26,27,28,29,30,31,32,33,34,35].

Phosphorus is an essential element in life, found in many biogenic molecules (DNA, RNA, and adenosine triphosphate (ATP)). Phosphorus is also found in many other important biomolecules, including cell membrane phospholipids such as sphingomyelin. There are also many examples of active pharmaceutical ingredients (APIs) containing phosphorus with important applications in the treatment of different afflictions. Consequently, a growing interest in the synthesis of phosphorus compounds has occurred in recent decades. One of the main goals of the researchers involved in this field was to conduct these syntheses under green conditions. Therefore, green syntheses in the field of phosphorus compounds were developed significantly using ultrasounds, microwaves, green solvents, sol-gel, and others. When ultrasounds (US) or microwaves (MW) were used, an increase in the yield was observed [26,27,28,29,30,31,32]. 

**Figure 2:**
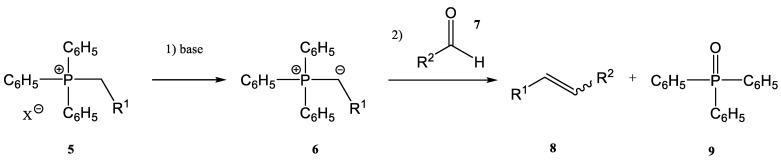
A general scheme of the classic Wittig reaction [9].

As already mentioned, one common synthetic route for the synthesis of alkenes is the Wittig reaction (together with its modified versions, as Horner–Wittig and aza-Wittig processes). First described in 1954, the Wittig reaction [1,2,3,4] has two main steps:

- the first step is the deprotonation reaction of a phosphonium salt (**5**) to obtain a phosphorous ylide (**6**)
- the phosphorous ylide (**6**) reacts with a compound containing a carbonyl group, an aldehyde (**7**), or a ketone to give the corresponding alkene (**8**) and phosphine oxide (**9**) (Figure 2)

Obtaining alkenes is of great interest to researchers because alkenes could be used further as reagents for several chemical syntheses in coupling reactions and asymmetric transformations, hydrogenation, cyclopropanations, cycloadditions, epoxidations, diol formation, and so on. Most research on the Wittig reaction has been focused, especially in recent years, on triphenylphosphine-derived phosphonium salts [6]. Non-stabilized ylides generally have an alkyl group as the side chain. Under lithium-salt-free conditions, these ylides showed a significant Z-selectivity [3,4,11,12]. Ylides that were stabilized with neighboring vinyl or aryl groups showed rather low selectivity and usually lead to the formation of mixtures with E and Z isomers [6,23,24,25,26].

Triphenylphosphine-derived ylides are the most common phosphorous reagents involved in the Wittig reaction. The synthesis of E-alkenes, starting from non-stabilized or semi-stabilized triphenylphosphoranes, is not sustainable by using the standard Wittig process. In this case, if used, the Wittig synthesis requires several modifications. If the phenyl substituents from a triphenylphosphorane were replaced with short-chain alkyl substituents (i.e., ethyl, propyl) with lower hydrophobic character, a significant increase in E-alkene isomer production was observed [36,37,38]. Thus, the non-stabilized and semi-stabilized ylides derived from trialkylphosphines showed high E-isomer selectivity when used in the Wittig reaction [6]. More recently, the Wittig reaction proved to be very useful in the field of organocatalysis [39,40]. Compounds as styrenes, dienes, vinyl ethers, or allenes, synthesized by using the Wittig reaction, are used in organocatalysis [6]. Organocatalysis includes a variety of chemical transformations and is used to describe any process that is facilitated by the use of a non-metallic organic catalyst [41,42,43,44,45,46].

The Wittig synthesis could be conducted using ultrasound in order to increase the interface area (and therefore the contact) between the reagents (i.e., ylide and aldehyde or ketone). Classic Wittig methods [1,2,3,4] are usually performed at low temperatures using strong bases. The ultrasound plays the role of a solvent by increasing the mixing process. In addition, the effects of the sonic waves are higher when the reaction is performed in small channels (diameter from 10 μm to 100 μm) than in a standard flask [47].

**Figure 3:**
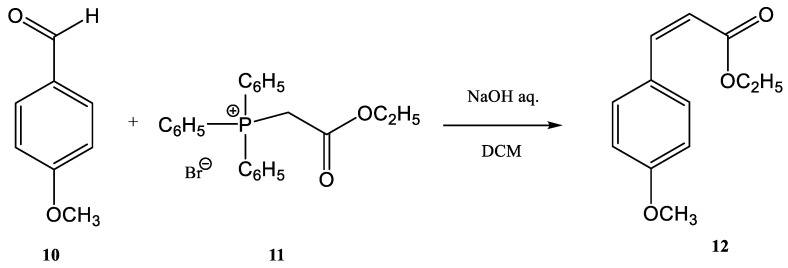
Synthesis of ethyl 4-methoxy-ethyl-cinnamate [47].

For example, the synthesis of several cinnamic esters and their derivatives using the Wittig reaction is of great interest because such compounds are further employed in several chemical industry areas of great interest (flavors, synthetic dyes, perfumes). Moreover, cinnamic moiety is found in many biologically active molecules. From this class of compounds, 4-methoxy-ethyl-cinnamate **12** (Figure 3) is a monoamine oxidase inhibitor. Monoamine oxidase inhibitors were the first type of antidepressant medication developed [47,48].

It was observed that cinnamic esters are synthesized easier, faster, and with a higher yield when the entire reactor is immersed in an ultrasonic bath. Several methods for the synthesis of this class of compounds have been published [47,48,49,50,51,52,53]. One example is the reaction of anisaldehyde **10** with (ethoxycarbonylmethyl)-triphenylphosphonium bromide **11** for obtaining ethyl 4-methoxy-ethyl-cinnamate **12** (Figure 3) [47,53].

El-Batta et al. proved that water is an effective environment for Wittig reactions by using stabilized ylides and aldehydes [54]. P-anisaldehyde slowly reacts with the ylides to obtain a mixture of E/Z cinnamic ester isomers after four hours at 20 °C, with a yield of 66% at a ratio E/Z 92/8. When the temperature of the reaction increased to 90 °C, after 30 min the yield of cinnamic ester increased to 90% without affecting or changing the E/Z-ratio. The water efficiency in the reaction environment in comparison with organic solvents is obvious, as the same reaction has been reported in refluxing DCM (four hours, 8% yield), in refluxing benzene (two days, 73% yield), and in ionic liquids at 60 °C (three days, 82% yield) [55,56,57]. On the other hand, when this synthesis procedure was performed in an ultrasonic bath, the product was obtained with a 70% yield in a shorter time. The protocol can be applied to different aldehydes, alkyl phosphonium salts, and bases for the Wittig synthesis of E-cinnamic esters under ultrasound in moderate to very good yields in the absence of any other phase transfer catalyst and in a shorter reaction time [47].

**Figure 4:**
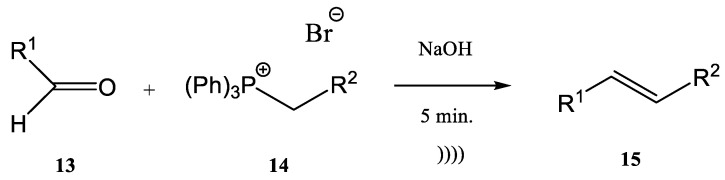
Ultrasound-assisted Wittig olefination in biphasic media [47].

The Wittig synthesis (sometime called the Wittig olefination) is one of the most famous phosphine-based reactions. The development of continuous flow processes for Wittig olefination reactions has undergone intensive study in recent decades, with the aim of increasing the yield. The combination of flow chemistry with microwave irradiation or ultrasonication opens a new perspective from this point of view. Many pharmaceutical compounds were synthesized through the Wittig olefination [58] by using a combination of ultrasound technology and continuous flow [59,60,61]. Riccaboni et al. [47] developed a catalyst-free continuous flow biphasic system for the Wittig synthesis of disubstituted alkenes **15** (Figure 4) [62,63].

The synthesis was performed starting from an aldehyde (**13**), triphenyl-phosphonium bromide (**14**), and NaOH at a ratio of the used reagents **13**:**14**:NaOH of 1:2:5 (an excess of phosphonium bromide **14** and of NaOH was used). The reaction mixture was immersed in an ultrasonic bath for enhancing the interfacial interactions in the absence of a phase-transfer catalyst (Figure 4) [47,62,63,64]. 

**Figure 5:**
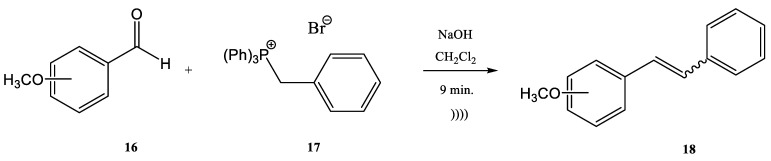
Continuous flow process for the synthesis of stilbene derivatives **18** by Wittig reaction with a benzyltriphenyl-phosphonium bromide salt **17** acting both as reactant and phase-transfer catalyst [65].

Modest to quantitative yields were obtained at room temperature after five minutes. The authors reported the in situ preparation of the phosphonium salt **14** by mixing triphenylphosphine (PPh_3_) and ethyl 2-bromoacetate. When the phosphonium salt **14** was prepared in situ, the yields of the synthesis increased [27]. A similar Wittig synthesis was proposed by Krajnc et al. [65]. Benzyl-triphenyl-phosphonium bromide salt (**17**) and *o*- or *p*-methoxy-benzaldehydes (**16**) were mixed at a 1:1 eq. ratio in CH_2_Cl_2_ and injected together with an aqueous solution of 0.1 M NaOH. The corresponding stilbene derivative **18** (Figure 5) was obtained after a maximum reaction time of 9–10 min. The yields changed from 68% for *o*-methoxybenzaldehydes to 90% for *p*- methoxybenzaldehydes.

**Figure 6:**
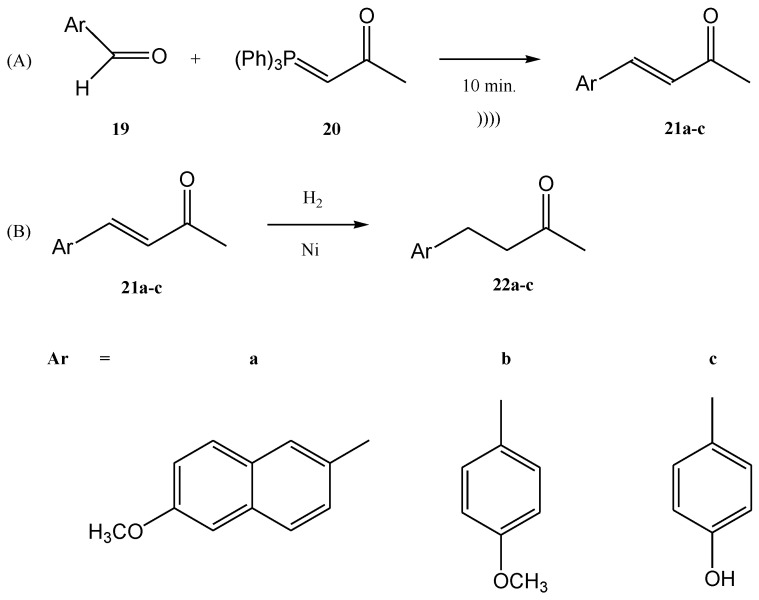
(**A**) The continuous flow Wittig olefination (**B**) the further reduction of the compounds **21a**-**c** [66].

Viviano et al. synthesized different active pharmaceutical ingredients using a Wittig olefination process [66]. Starting from the aldehyde **19**, different synthetic routes were used in order to synthesize the 4-aryl-3-buten-2-one intermediates 2**1a**–**c**. The reaction can be conducted as a continuous flow process (Figure 6A) as follows: aldehyde **19** reacts with (acetylmethylene)triphenyl-phosphorane **20** in DMF and the products 4-aryl-3-buten-2-one intermediates **21a**–**c** in good yields (around 98%) at 210 °C for 10 min. Then, 4-aryl-3-buten-2-ones **21a**–**c** were further converted under pressure using a Raney-Ni catalyst through a hydrogenolysis reaction (Figure 6B) [27]. The hydrogenolysis represents a chemical reaction where a carbon–carbon or carbon–heteroatom bond is cleaved or undergoes “lysis” by hydrogen.

The hydrogenolysis reaction of the alkene **21a** takes place in ethanol and the hydrogenolysis process of alkenes **21b** and **21c** takes place in DMF. At temperatures ranging from 20 °C to 100 °C, the final products with active pharmaceutical properties were obtained in good yields, as follows: **22a**—91%, **22b**—90%, **22c**—94%. The compounds **22a** and **22b** are commonly used in cosmetics. On the other hand, the compound **22c**, currently named nabumethone, is a nonsteroidal anti-inflammatory drug used to reduce pain, swelling, and joint stiffness from arthritis. Nabumethone can be used only with a doctor or pharmacist’s recommendation [66]. 

**Figure 7:**
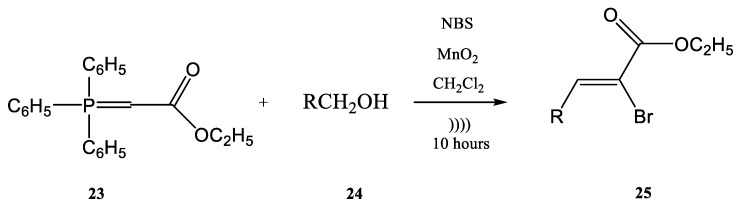
The tandem halogenation–oxidation–Wittig reaction in one-step, conducted under ultrasonication [67].

The previously discussed examples showed the Wittig reaction employed alone on different syntheses. Moreover, the Wittig reaction could be involved in the synthesis of phosphorus compounds in tandem with other types of chemical processes in a one-step procedure. For instance, the halogenation of an ylide and the oxidation of an alcohol with the common reagent MnO2 as the oxidant and a Wittig reaction together could be conducted in a one-step procedure using ultrasounds. In the work published by Karama et al. [67] the (carboethoxymethylene)triphenyl-phosphorane **23** reacted with a reactive alcohol (as for instance aromatic, allylic and propargylic alcohols) in the presence of *N*-bromosuccinimide (NBS) and manganese dioxide, in CH_2_Cl_2_ (Figure 7).

When a reactive alcohol was used, a mixture of Z- and E-isomers (Table 1) of the α-bromo-α,β-unsaturated esters **25a**–**g** were obtained by the one-step halogenations–oxidation–Wittig chemical process in good yields (around 90%). 

On the other hand, when low-reactive alcohols were employed, such as alkanols, the obtained yields decreased significantly [67]. An example shown in Table 1 includes the use of octanol, which gave a yield of only 21%. For this one-step synthesis involving a Wittig reaction, 10 mmol of manganese dioxide was added to a solution containing 1.4 mmol of N-bromosuccinimide (NBS), 1.3 mmol of (carboethoxymethylene)triphenylphosphorane, and 1 mmol alcohol in 12 mL of CH_2_Cl_2_ as solvent. The resulted mixture was further sonicated for 10 h and the products were obtained with the yields and Z:E ratios shown in Table 1 corresponding to the used reagents [67].

**Figure 8:**
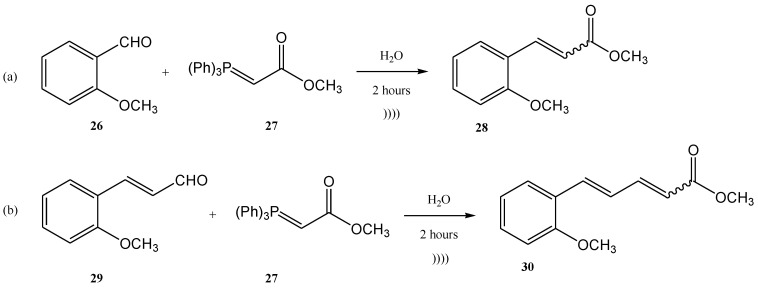
Examples of two Wittig reactions used as green methods, performed under ultrasound [68].

Another example of using the Wittig reaction as a green method (in this case also the use of ultrasounds) using phosphonium ylide as reagent is the work of Maity et al. [68]. The Wittig process was performed under ultrasonication, starting from aldehydes (**26**, **29**) and ylides (**27**). The products **28** and **30** were further obtained with high yields as a mixture of E- and Z-isomers (E/Z = 76/24 in the case of the product **28**, and 84/16 in the case of the compound **30**) [68] (Figure 8a,b).

**Figure 9:**
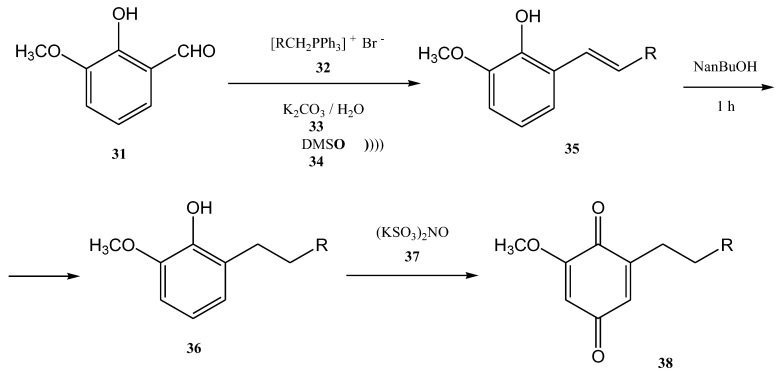
The Wittig synthesis of 2-methoxy-6-alkyl-1,4-benzoquinones, performed under ultrasonication [64].

The ultrasound irradiation for the Wittig reaction is usually performed in a water bath of an ultrasonic cleaner with a frequency of approximately 40 KHz and a power of approximately 250 W [69]. Currently, several products of growing interest are synthesized in this way. Benzoquinones, for instance, represent an important class of biologically active compounds, which were also obtained by the Wittig reaction performed under ultrasound. [64,69] 2-methoxy-6-alkyl-1,4-benzoquinones are compounds that occur in nature (usually in plants) and most of them have significant biological activity (anti-cancer activity and 5-1ipoxygenase inhibitory activity). Lipoxygenase enzymes catalyze the deoxygenation processes of polyunsaturated fatty acids to obtain lipids. The ultrasound-assisted Wittig reaction of *o*-vanillin **31** with alkyltriphenyl phosphonium bromides **32** in the presence of K_2_CO_3_ leads to the formation of styrene **35** in 72–81% yields (Figure 9) [5,64]. 

This reaction requires a mixture of DMSO (**34**) and water as solvents at 90–100 °C. Then, the2-methoxy-6-alkenyl-1,4-benzoquinones can be obtained through the hydrogenolysis reaction. If the hydrogenolysis of the styrene **35** is performed directly to obtain 2-methoxy-6-alkenyl-1,4-benzoquinone, the double bond from its structure is actually reduced. Consequently, the styrene **35** was first treated with metallic sodium in *n*-butanol. In this way, the 2-methoxy-6-alkyl-phenols **36** were further successfully synthesized in 74–84% yields after one hour at 80–90 °C. The conjugated olefin was reduced but the isolated olefin was not affected [5,64].

This step of the synthesis was followed by the oxidation of 2-methoxy-6-alkylphenols **36** with Fremy’s salt (KSO_3_)_2_NO (**37**). Then, 2-methoxy-6-alkyl-1,4-benzoquinones **38** was obtained as a solid yellow product in 79–92% yields [64].

## 3. Aza-Wittig Reactions Performed under Sonication Conditions

As previously mentioned, the aza-Wittig synthesis is a modified Wittig reaction that leads to products containing C=N bonds. In recent years, the aza-Wittig reaction has attracted a lot of interest because it has showed huge potential for the synthesis of a large variety of phosphorus- and nitrogen-containing heterocycles [19,70,71,72,73]. Such heterocyclic compounds could be used further in the synthesis of functionalized iminophosphoranes. The existence of nucleophilicity in the nitrogen atom made the use of these iminophosphoranes possible as aza-Wittig reagents. Iminophosphoranes are important reagents in organic chemistry syntheses, especially for obtaining different compounds with biological and pharmacological activity [19,74,75,76,77]. For example, compounds including 1,3,4-oxadiazole structures showed important pharmacological and therapeutic activities (anti-inflammatory and hypotensive effects) [78,79].

**Figure 10:**
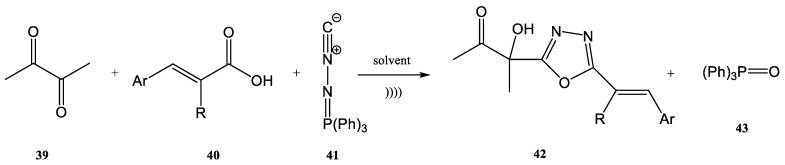
Synthesis of substituted 1,3,4-oxadiazole derivatives under ultrasonic irradiation [77].

A simple and efficient example of using ultrasound for the synthesis of heterocyclic compounds through the aza-Wittig process is the preparation of substituted 1,3,4-oxadiazole derivatives (Figure 10) [77,80,81]. 

The condensation of biacetyl **39**, 4-methylcinnamic acid **40,** and *N*-isocyaniminotriphenyl-phosphorane **41** was carried out in several solvents (CH_2_Cl_2_, DMF, THF, CH_3_CN, 1,4-dioxane and EtOH) under ultrasound at room temperature. *N*-isocyan-iminotriphenyl-phosphorane (1 mmol), biacetyl (1 mmol), and *E*-cinnamic acid (1 mmol) were mixed with 15 mL of solvent (one of the above-mentioned solvents). The obtained reaction mixture was then placed in an ultrasonic bath with a power of 100 W. The method to obtain fully substituted 1,3,4-oxadiazole derivatives (**42**) under ultrasound offered important advantages of faster reaction rates, higher yields, and nevertheless higher purity of the product, in comparison with the classic stirring methods (even if the classic synthesis was performed at higher temperature) [77]. Under stirring conditions, after 12 h the product **42** was obtained at a 90% yield. On the other hand, under ultrasonication by using an ultrasonic bath of 100 W, the product **42** was obtained at a 97% yield in only 16 min. Therefore, a small increase in the reaction yield was observed under ultrasonication. In addition, the main advantage was a decrease in the time necessary to complete the synthesis (from 12 h to only 16 min). The power of the ultrasonic bath also favorably influenced the results. The yield obtained for the use of an ultrasonic irradiation of 100 W for 16 min was better than the yield observed for the use of an ultrasonic irradiation of 150 W for the same period of 16 min [77].

**Figure 11:**
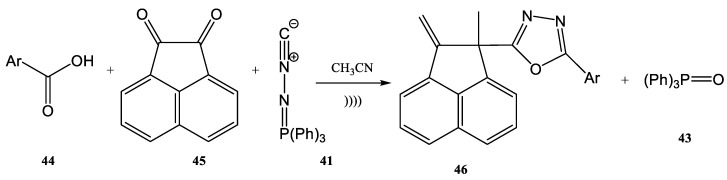
Synthesis of disubstituted 1,3,4-oxadiazole derivatives (**46**) under ultrasound irradiation. [82].

In another report by Rouhami et al. [82], disubstituted 1,3,4-oxadiazole derivatives (**46**) were successfully synthesized using the aza-Wittig reaction under ultrasound irradiation (Figure 11). The synthesis conducted under ultrasound can be defined as a chemical synthesis performed in a liquid medium in the presence of pressure waves.

The carboxylic acid **44** (1 mmol), acenaphthoquinone **45** (1 mmol), (N-isocyanimino)triphenylphosphorane **41** (1 mmol), and CH_3_CN (10 mL) as solvent were mixed together. The resulted mixture was irradiated by ultrasound using an ultrasonic bath at 100 W. During ultrasound irradiation, the temperature was kept at approximately 25 °C by cooling in an ice bath. The yields of the synthesis in Figure 11 show an increase from around 80% to a maximum 93%, and the reaction time decreased from 24 h to only 15 min when ultrasounds were used [82].

**Figure 12:**
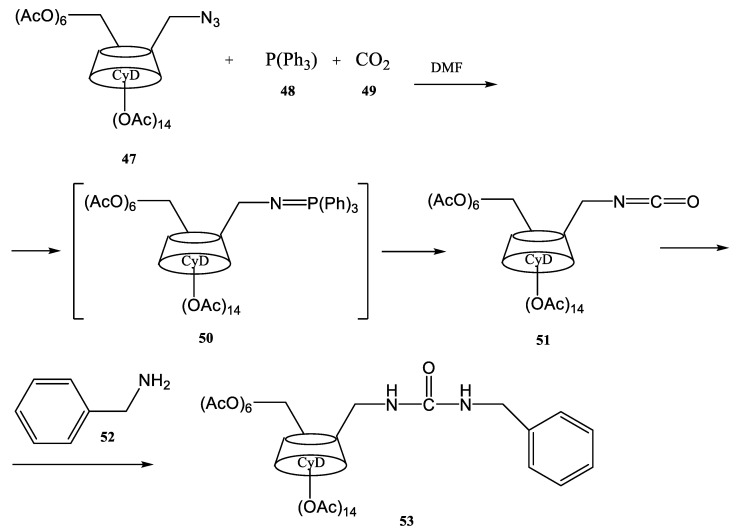
The Staudinger–aza-Wittig reaction used to obtain cyclodextrin derivatives [83].

Another study of the ultrasonication effect on a modified Wittig synthesis (Staudinger–aza-Wittig reaction) was reported in the work published by Scondo et al. [83]. The Staudinger reaction represents a reduction in organic azides yielding the corresponding primary amines. The reaction was developed by Hermann Staudinger in 1919. The synthesis reported in the work of Scondo et al. (Figure 12) is related to cyclodextrins, an important class of cyclic oligosaccharides containing a macrocyclic ring of glucose units inter-connected by 1,4 glyosidic bonds [83].

Cyclodextrins (CyDs) are used in the food industry, in different chemical industries, in agriculture and environmental engineering, and as pharmaceutical products in drug-delivery systems. As a consequence, CyDs have attracted a growing interest in recent years due to their various applications in these important research fields [84]. The regioselective functionalization of their hydroxyls groups strongly improved their catalytic activities at the supramolecular level. Recent papers compared several CyDs functionalization carried out both under conventional conditions and under ultrasonication [85,86,87,88,89,90,91,92]. The results showed a significant improvement in the yields and in the reaction times. Isocyanate and urea formation in a Staudinger–aza-Wittig reaction takes place better with higher yields under ultrasonication. In the synthesis by a Staudinger–aza-Wittig reaction of different cyclodextrins derivatives performed by Scondo et al., the ultrasound source was a Bandelin-HD2070 generator (20 KHz, 70 W). The 6A-azido-6A-deoxy-per-O-acetylated-β-cyclodextrin **47** was treated with triphenylphosphine **48** in the presence of CO_2_ as the electrophile and benzylamine **52** as the nucleophile in anhydrous DMF. The 6A-benzylureido-6A-deoxyper-O-acetyl-β-cyclodextrin **53** was obtained in a shorter time and in an excellent yield in comparison with the synthesis conducted in classic conditions [83].

## 4. Wittig–Horner Reactions Conducted Using Ultrasound

The Horner–Wardsworth–Emmons (HWE) method is a chemical reaction between a phosphonate carbanion and a carbonyl compound (as aldehydes or ketones) to produce mostly E-alkenes [3,4,91,93,94,95,96,97,98,99,100]. Leopold Horner published a modified Wittig reaction in 1958 using phosphonate-stabilized carbanions as reagents [91,93]. William Wardsworth and William Emmons added further changes to this procedure [91,94]. It is named the Horner–Wadsworth–Emmons (HWE) or the Wittig–Horner synthesis. In contrast to phosphonium ylides used in the classic Wittig reaction, the phosphonate-stabilized carbanions are more nucleophilic but less basic. The novelty and the advantage of HWE synthesis is that the phosphonate-stabilized carbanions can be alkylated. Unlike phosphonium ylides, the dialkylphosphate salt could be easily removed and separated by an aqueous extraction procedure.

**Figure 13:**
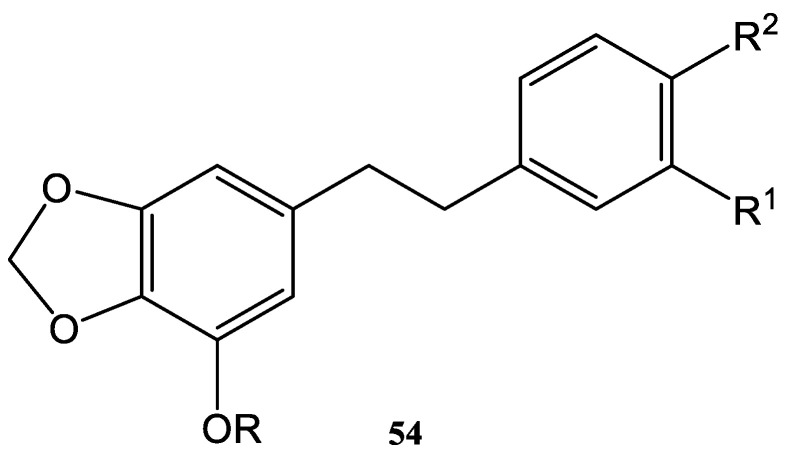
Structures of different dihydrostilbenes (**54**) [101].

In recent decades, the positive effects of using ultrasound in order to develop green syntheses has been studied and applied to the Horner–Wardsworth–Emmons process (HWE). For instance, different dihydrostilbenes were synthesized by Wittig–Horner chemical syntheses under ultrasound. Dihydrostilbenes (Figure 13 and Table 2) represent an important class of natural products of great interest.

Due to their pharmacological effects, anti-oxidant activities [85], cancer preventive effects [86], anti-tumor activities [87], inhibition of cyclooxygenase [88], and inhibition of platelet aggregation [89,90,91], dihydrostilbenes are involved in many applications of great interest. Cyclooxygenase (COX), also known as prostaglandin-endoperoxide synthase, is an enzyme responsible for the formation of prostanoids (as tromboxane) and prostaglandins (as prostacyclin) from arachidonic acid. Prostanoids are active lipid mediators that regulate inflammatory response. The prostaglandins are physiologically active lipid compounds. Prostaglandins have hormone-like effects and are found in almost every tissue in humans. Every prostaglandin contains 20 carbon atoms (including a 5-C ring). In addition, some synthetic dihydrostilbenes (Figure 13, Table 2) showed strong anti-mitotic activity in a broad spectrum of human cancer lines [90]. Moreover, nucleoside-analogous compounds, such as N-glycosylated 4-halomethyl 1H- or 2H-1,2,3-triazoles, are of great interest as radiomimetic substances, bactericides, and viricides, respectively [102].

**Figure 14:**
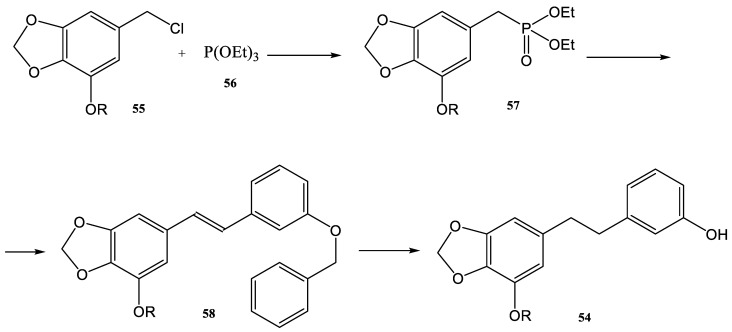
Synthesis route of natural dihydrostilbene compounds **54a**,**b** (if R is CH_3_ the product is **54a** and if R is H the product is **54b**) [101].

As a consequence, the development of a synthesis with high yield and high selectivity, in mild and green conditions, in a shorter reaction time, and with different dihydrostilbenes was an important goal for the researchers in this field in recent decades. The method used for the synthesis of the compounds **54a** and **54b** is described in Figure 14.

Benzyl chlorides **55** are precursors of dihydrostilbenes **54a** and **54b**. The first step for the synthesis of those stilbene derivatives is the Michaelis–Arbuzov reaction of **55** with P(OEt)_3_ (**56**). This reaction provided the intermediate compound **57**, which could be then used directly in the next step (Figure 14) in the Wittig–Horner reaction. The products of the Wittig–Horner reaction (**58**) were converted further by a catalytic hydrogenation to the natural dihydrostilbenes **54a** and **54b,** with 94.5% and 98.3% yields, respectively. These stilbenes were obtained from the reduction of the double bond and the removal of the benzyl protecting group (Figure 14) [101].

**Figure 15:**
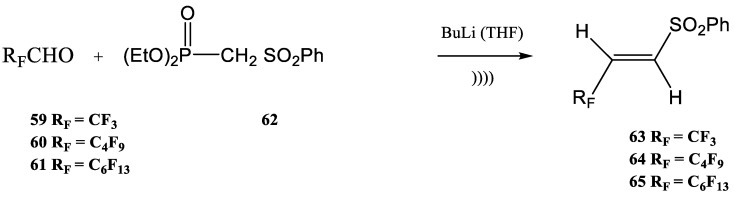
The synthesis of (E)-1-perfluoroalkyl-2-phenylsulfonyl-ethenes (**63**–**65**) by an ultrasound-assisted Wittig–Horner olefination [105].

Furthermore, different biologically active fluorinated 1,2,3-triazoles were synthesized by the Wittig–Horner method under ultrasound [92,93,94,95,96,97,98]. The main step of the fluorinated 1,2,3-triazoles synthesis is the 1,3-dipolar cycloaddition. Perfuoroalkyl-substituted vinyl sulfones are used in the syntheses of different fluorinated heteroaromatic compounds by 1,3-cycloaddition. Therefore, (E)-1-perfluoroalkyl-2-phenylsulfonyl-ethenes **63**–**65** were synthesized by an ultrasound-assisted Wittig–Horner olefination from the perfluoroalkanals **59**–**61** and the phosphonate **62** (Figure 15) [103,104,105].

As fluoral CF_3_CHO (**59**) is gaseous, the procedure for the preparation of **63** was slightly modified in comparison with the syntheses used for obtaining compounds **64** and **65**. When the syntheses were performed under ultrasound, the reaction time decreased significantly. For example, compound **63** was synthesized from fluoral at a temperature of -78ºC after three hours. At the same time, the other two products (**64** and **65**) were obtained under sonication in only 30 min. All three compounds **63**–**65** were synthesized by Wittig–Horner olefination in moderate yields (51–58%). The sonication was obtained with an ultrasonic bath Vibracell VCX-400 at a frequency of 20 kHz with a power of 120 W.

**Figure 16:**
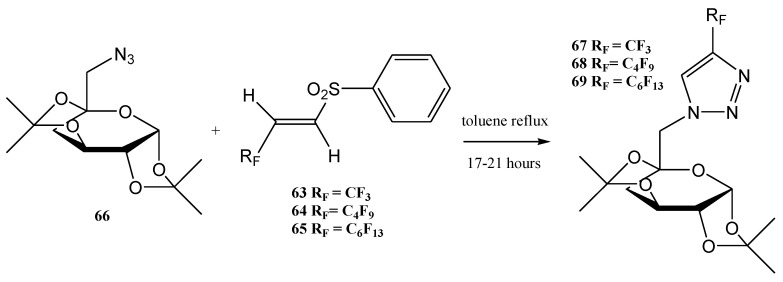
The synthesis of perfluorinated triazole derivatives by the 1,3-dipolar cycloadditions of the azides with vinyl sulfones [105].

The compounds **63**–**65** were used further for the synthesis of 4-perfkuoroalkyl-substituted 1,2,3- triazoles **67**–**69** (an example is the 1,3-dipolar cycloaddition of the azide **66** with the homologous vinyl sulfones **63**–**65** in refluxing toluene). Only a single regio-isomeric 1,2,3-triazole derivative was formed in this reaction (Figure 16) [105].

The reversed nucleosides **67**–**69** (i.e., 4-perfluoroalkyl-substituted 1,2,3-triazoles linked to the C-atom 6 of D-galactose and D-altrose) were synthesized by 1,3-dipolar cycloadditions using the monosaccharide azide **66** and the perfluoroalkyl-substituted phenyl vinyl sulfones **63**–**65**, at yields of 72–75% (Figure 16). Various natural antibiotics contain carbohydrate moieties with amino-deoxy structures, especially heterocycles linked to sugars. These compounds represent an interesting group of mimetic products, such as the examples of 1,2,3-triazole derivatives synthesized in the work published by Hager et al. [105,106,107,108].

**Figure 17:**
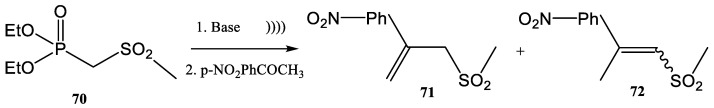
Example of carbon–carbon bond formation by a Wittig–Horner reaction conducted under ultrasound [90,109].

A Wittig–Homer synthesis between sulfonomethyl-phosphonate (**70**) and p-nitroacetophenone (p-NO_2_PhCOCH_3_) was reported in [109]. The synthesis was performed under ultrasonic irradiation (Figure 17) [90].

They observed that the use of ultrasound helped the synthesis through a significant increase in yield for the obtained product, vinyl sulfone (**72**). When the Wittig–Horner synthesis is applied starting from ketones, the yields are rather low. However, by using ultrasound for two hours, the vinyl sulfone (**72**) was obtained at a higher yield (Table 3).

The authors showed that the E-/Z-isomerism observed is related to ultrasonic cavitation since pure E-vinyl sulfone is 20% isomerized by sonication in THF [90,103,109]. Such isomerization reactions have never been observed in sonochemistry, except under catalysis. When ultrasounds were used for the Wittig–Horner synthesis plotted in Figure 17 using BuLi as base, the overall yield increased from 33% to 48%, but the ratio E/Z decreased. If NaH was used as base instead of BuLi, the ratio E/Z of the compound **72** was improved (80:20) and the overall yield increased even more, up to 66%. The **72**:**71** and E/Z ratios were determined by ^1^H NMR spectroscopy [109]. The authors commented that sonication is well known to promote single electron transfers in solutions and non-stabilized ylides can react via a non-ionic pathway. On the other hand, stabilized ylides react under sonication via transient radical species under heterogeneous catalysis [90].

**Figure 18:**
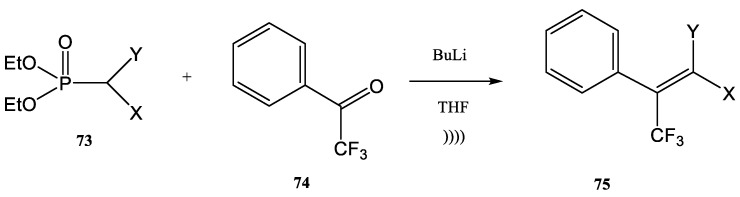
Wittig–Horner synthesis of trifluoromethylated alkenes under ultrasound [32,92,103].

As an example of reactions of perfluoroalkylated building blocks, the synthesis of trifluoromethylated alkenes should be mentioned (Figure 18 and Table 4).

The products **75** are synthesized in a Z/E-mixture by the Wittig–Horner method, as shown in Table 4, (Figure 18), starting from trifluoroacetophenone **74** and different phosphonates **73**, in THF and in the presence of BuLi. The yields increased from 50% to 63% function of the substituents X and Y (Table 4) [32,92,103].

## 5. Conclusions

The Wittig reaction is one of the most powerful and attractive methods for the construction of various alkenes. The Wittig reaction is one of the most useful reactions for the synthesis of olefins. In the last few decades, this reaction has been extensively studied and employed in synthesis on an industrial scale. The so called “enabling techniques”, mainly non-conventional energy sources such as microwaves (MW) [30,31,53,61,88,109] and ultrasound (US) [22,32,46,47,59,60,61,62,63,64,77,78,79,80,81,85,86,87,88,89,90,92,101,102,105,110,111,112], can significantly improve the reaction yields in organic synthesis. Moreover, by using ultrasound in Wittig reactions and their modified versions for different organic syntheses, the E/Z ratio of the obtained products can be controlled. The E/Z ratio of the synthesized compound was determined by ^1^H NMR spectroscopy.

Ultrasound-promoted synthesis has attracted much attention during the past few decades as a green synthetic path. The syntheses performed under ultrasound are faster due to the formation, growth, and collapse of acoustic bubbles in the reaction medium. The use of ultrasound helps those Wittig syntheses described in the present review by shortening the reaction time and by increasing the yield of products.

## Figures and Tables

**Table 1:** One-pot synthesis of unsaturated esters **25a**–**g** [67].

R	Product	Yield (%)	Z:E Ratio
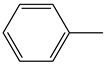	**25a**	85	94:6
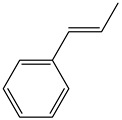	**25b**	81	95:5
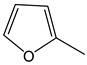	**25c**	91	89:11
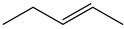	**25d**	90	100:0
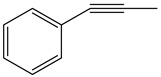	**25e**	86	92:8
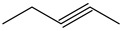	**25f**	87	76:24
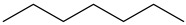	**25g**	21	90:10

**Table 2:** The radicals R, R_1_, and R_2_ from the structure of dihydrostilbenes **54a**–**k**.

Dihydrostilbenes	R	R_1_	R_2_
**54a**	CH_3_	OH	H
**54b**	H	OH	H
**54c**	CH_3_	NH_2_	H
**54d**	H	NH_2_	H
**54e**	CH_3_	H	OH
**54f**	H	H	OH
**54g**	CH_3_	H	F
**54h**	H	H	F
**54i**	H	H	OCH_3_
**54j**	H	H	N(CH_3_)_2_
**54k**	CH_3_	OCH_3_	OH

**Table 3:** Conditions, 72:71 ratio, overall yield, and E/Z ratio of the compound **72**.

Base	Conditions	Ratio 72:71	Ratio E/Z	Yield (%)
BuLi	Stirring	95:5	75:25	33
BuLi	Ultrasound	85:15	60:40	48
NaH	Ultrasound	60:40	80:20	66

**Table 4:** The substituents X and Y used in the structure of the phosphonate reagent **73** and found on the structures of the products **75** obtained from the Wittig–Horner synthesis showed in Figure 18.

X	Y	Yield %	Z:E
SO_2_CH_3_	H	54	61:39
SO_2_C_6_H_5_	H	63	43:57
OCH_3_	CN	50	58:42

## Data Availability

Not applicable.
